# Animal Models of Febrile Seizures: Limitations and Recent Advances in the Field

**DOI:** 10.3390/cells13221895

**Published:** 2024-11-16

**Authors:** Alexandra V. Griflyuk, Tatyana Y. Postnikova, Aleksey V. Zaitsev

**Affiliations:** Sechenov Institute of Evolutionary Physiology and Biochemistry of Russian Academy of Sciences, 44, Toreza Prospekt, Saint Petersburg 194223, Russia; griflyuk.al@mail.ru (A.V.G.); tapost2@mail.ru (T.Y.P.)

**Keywords:** epilepsy, rat, hippocampus, animal model, hyperthermia

## Abstract

Febrile seizures (FSs) are defined as seizures occurring in children aged 6 months to 5 years with a background of elevated body temperature. It is one of the most common neurological disorders of childhood, emphasizing the importance of understanding the causes of FSs and their impact on the developing nervous system. However, there are significant limitations to the technologies currently available for studying the etiology and pathophysiology of seizures in humans. It is currently not possible to adequately capture the subtle molecular and structural rearrangements of the nervous system that can occur after seizures in humans. The use of animal models can be invaluable for these purposes. The most commonly used models in modern research are hyperthermic models in rats and mice aged 10–12 days. While these models can reproduce many of the characteristics of FSs, they have certain limitations. This review outlines the key considerations when working with models of FSs, provides an overview of current approaches to producing seizures in different model subjects, and presents a summary of key findings regarding morphological and functional changes in the brain and behavioral alterations that have been identified in studies using animal models of FSs.

## 1. Introduction

Febrile seizures (FSs) are a common neurological disorder affecting children between the ages of 6 months and 5 years, with a peak incidence during the second year of life [1]. The precise mechanism underlying FSs remains unclear. It presents when body temperature exceeds 38 °C in the absence of a discernible inciting factor, such as trauma, infection, or intoxication of the central nervous system [1]. Epidemiological studies have demonstrated that the prevalence of FSs varies according to geographical region. The highest incidence of FSs in children has been observed in Guam (11%) [2] and East Asian countries (8–11%) [3,4], whereas, in the USA and Europe, the incidence of FSs ranges from 2 to 5% [5,6,7,8].

FSs are more frequently reported during the cold season, which corresponds to the peak of febrile illnesses in children [9]. The most common etiologies of fever in the context of FS development are viral infections, including influenza, adenovirus, parainfluenza, and herpesvirus-6 [10,11,12]. Among bacterial infections, FSs most frequently manifest in the context of Otitis media [12,13]. In children with a genetic predisposition to epilepsy, a fever resulting from vaccination may precipitate the development of FSs [14]. Seizures are more likely to occur within an hour of the onset of fever, but a more significant factor in assessing the risk of FSs is the height of the fever rather than the rate of temperature rise [15,16].

There is still no consensus on whether FSs cause brain damage and the development of neurological disorders later in life. A large cohort studies revealed a 1.4-fold increased risk of schizophrenia [17] and a 1.3-fold increased risk of attention deficit hyperactivity disorder (ADHD) [18]. Additionally, boys with a history of FSs demonstrated a higher risk of ADHD than girls [19]. A significant correlation between FSs and autism spectrum disorders has been documented [20]. Furthermore, it has been suggested that the correlation between mental illness and FSs is more pronounced in children with recurrent FSs and in those with an onset of FSs after three years of age [21]. However, an earlier age of FS onset (less than 12 months) and seizure duration exceeding 15 min are associated with an elevated risk of epilepsy [22,23]. However, the precise causal relationship between FSs at an early age and the subsequent development of neurological and psychiatric disorders remains unclear. It is conceivable that FS itself or its concomitant treatment may damage the developing brain, thereby predisposing individuals to mental illness at a later stage of life. However, it is possible that genetic factors or developmental abnormalities occurring during the prenatal and early postnatal periods and functional or structural alterations to the neural system may ensue, potentially culminating in both FSs and subsequent psychopathology.

It is crucial for clinical practice to have a solid understanding of the mechanisms underlying seizures. However, it is not possible to fully investigate the underlying causes of seizure development, their associated consequences, and to pursue a means to anticipate and avert the onset of seizures in humans. The main reason for this is that FSs typically emerge abruptly and seldom manifest in circumstances where medical supervision is feasible. Moreover, the current methods do not allow for the study of molecular and structural changes in living human nervous tissue. It is therefore essential to develop reliable models that accurately replicate the characteristics of seizures in a child to study the effects of FSs on the developing brain.

The original FS model, which involves raising the body temperature of immature rats using an infrared lamp, was first proposed in the early 1980s [24]. Subsequently, new methods for modeling hyperthermia, a condition that induces seizures in animals, were described using microwave radiation [25], hot air [26], and hot water [27]. The next significant advancement in the field of animal modeling of FSs was the initiative to develop a model based on fever rather than hyperthermia [28,29]. This approach more closely resembles the clinical characteristics of FSs. Additionally, the animal model, initially developed in rats, has been adapted for use in mice [30], *Danio rerio* [31], and *Drosophila* [32], opening new avenues for investigating genetic factors in the pathogenesis of FSs.

In this review article, we concentrate on several crucial criteria that must be considered when modeling FSs in animals. These issues were partially addressed in the review by Bender et al. (2004) [33]. This paper provides updated and expanded information on the key factors that need to be considered when working with an FS model, describes the existing approaches for seizure generation in different model subjects, and summarizes the main results obtained so far in studies with animal models of FSs.

## 2. Key Features That Should Reproduce the FS Model

### 2.1. The Main Types of FSs

Currently, FSs are classified as simple or complex, depending on the duration and the presence of recurrent episodes. Simple FSs last less than 15 min and occur no more than once a day. It is the most common form of seizure in children (about 70% of all reported cases). Complex FSs include seizures lasting more than 15 min or recurrent episodes over a 24 h period. The most severe type of complex FSs is febrile status epilepticus—a seizure lasting more than 30 min without the recovery of consciousness [1]. Cohort studies have demonstrated that the risk of developing temporal lobe epilepsy is significantly elevated in children who have experienced complex FSs in comparison to those who have experienced simple FSs [22,23]. This suggests that different types of FSs may exert disparate effects on the developing nervous system. Consequently, when modeling FSs in animals, it is imperative to meticulously control the duration of the seizures, thereby enabling an in-depth examination of the ramifications of simple and complex FSs, and febrile status epilepticus.

### 2.2. Age-Related Characteristics of FSs

**Figure 1:**
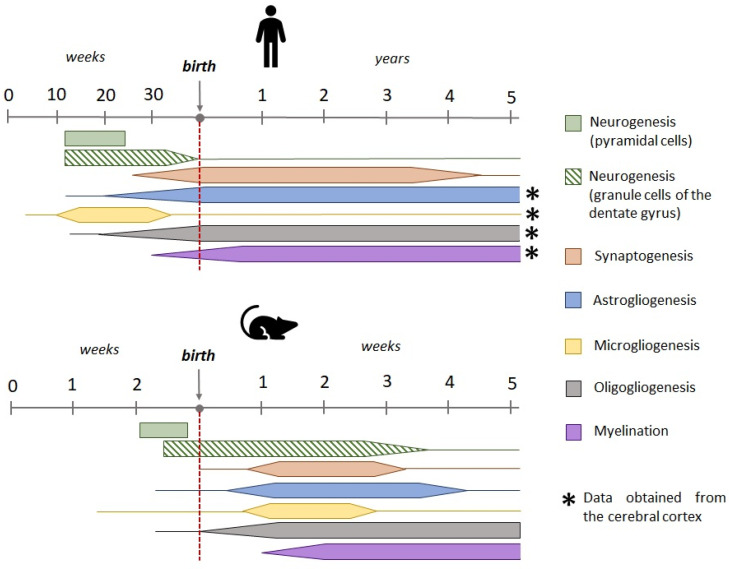
Dynamics of key developmental processes of the human and rodent hippocampus during prenatal and early postnatal development (except for gliogenesis and myelination in humans, these data are presented for the cerebral cortex) [36,40,41,42,43,44,45,46,47,48].

FSs in children occur mainly between the ages of 6 months and 5 years. Therefore, when modeling this type of seizure, it is important to consider the age of the animals, which should correspond to the period of greatest susceptibility to seizures in children. Early studies looked at the nervous system as a whole and, based on the rate of brain growth and the process of myelination, suggested that the 5–7-day-old rat might be comparable to a newborn human in terms of nervous system development [34,35]. More recent studies have compared the stages of maturation of individual structures, particularly the hippocampus and cerebral cortex, and these studies show that the first postnatal week of rat life is close to the third trimester of human prenatal development and the second postnatal week to the first year of human life [36,37]. There are notable discrepancies in the processes of neurogenesis and microgliogenesis in the rodent and human dentate gyrus (Figure 1). In humans, most granular cells in the dentate gyrus emerge by the 35th week of prenatal development. After this point, the rate of neurogenesis declines and maintains a consistent level throughout adulthood. In contrast, the main cohort of granular cells in the rat dentate gyrus emerges by the end of the first postnatal month [38]. Microglial cell progenitors appear at the earliest stages of prenatal development; however, in humans, microglial development and maturation occur prenatally and microglial cells acquire a mature phenotype by the end of prenatal development, whereas, in rats, microglia mature during the first weeks of postnatal development [39,40,41,42].

When assessing the susceptibility to hyperthermic convulsions in rats of different ages, it was found that the most appropriate age was 10–13 days of postnatal life, as this is the age at which convulsions occur at a temperature close to the threshold temperature for the development of FSs in children, with convulsions developing in most animals and occurring in a stereotypical pattern [26,49]. In rats younger than 10 days, the threshold temperature for seizure onset and the pattern of seizure are highly variable [26]. As rats mature, the threshold temperature increases. In rats aged 10–12 days, body temperature must reach 39–41 °C to develop seizures [26]; at 15 days of age, the threshold temperature is 43 °C [50]; and in rats aged three weeks, body temperature must exceed 44 °C to develop seizures [50], which is significantly different from the clinical manifestation of FSs in humans.

Thus, animals of suitable age should be used in models; for rats and mice, this is the middle of the second postnatal week.

### 2.3. Body Temperature

FSs develop at temperatures above 38 °C, so animal models should replicate this while avoiding extremely high temperatures (>42 °C) that are life-threatening. Therefore, body temperature should be monitored continuously when modeling FSs. It is not possible to measure brain temperature directly without implanting a temperature sensor in the nerve tissue, but it has been shown that the temperature curves of the brain and the core are similar [51]. For this reason, the rectal temperature of animals is monitored when FSs are modeled. If the body temperature rises above 41 °C, the animals should be placed on a cool surface to reduce the body temperature.

### 2.4. The Source of the Ictal Activity in the Brain During FS

Clinical case reports have noted edema and hippocampal damage in children following FSs [52,53,54]. This may suggest that the hippocampus is the source of the abnormal activity, but the unpredictable onset of FSs makes this very difficult to confirm. To our knowledge, a single EEG recording obtained directly during an FS in an 11-month-old child has been described, in which abnormal activity was recorded in the temporal regions [55]. The description of the behavior of children with FSs also suggests a hippocampal origin of the seizures [56].

In a hyperthermic model of FSs in 10-day-old rats, rhythmic activity was recorded in the amygdala and dorsal hippocampus concurrent with behavioral manifestations of seizures. At the same time, only rare non-rhythmic discharges were observed on EEG recordings from the cerebral cortex [26].

## 3. Factors That Influence the Course of FSs

### 3.1. Genetic Background

**Figure 2:**
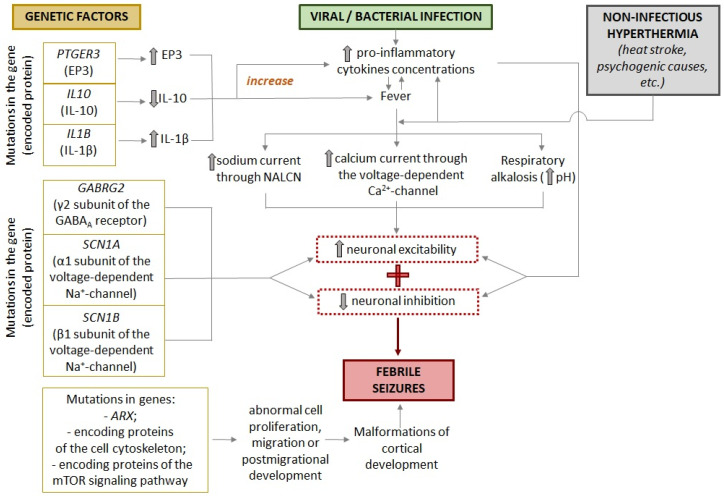
The etiology of FSs. The most common cause of FSs is a viral or bacterial infection, which results in the release of pro-inflammatory cytokines and fever. Both pro-inflammatory cytokines (Section 5.3) and elevated brain temperature (Section 5.2) result in enhanced neuronal excitability. An increase in blood pH, which is indicative of respiratory alkalosis, has been observed in individuals experiencing fever [57,58]. This phenomenon has also been linked to an elevation in neuronal excitability [59]. Specific genetic mutations may elevate the probability of developing FSs or increase the likelihood of FSs becoming more severe (Section 3.1). An elevation in body temperature that is not infectious in nature also results in the release of pro-inflammatory cytokines (Section 5.2). Cortical malformations have been identified as a potential cause of both FSs and epilepsy (Section 3.2) [60,61,62,63,64,65,66,67,68,69,70]. EP3—prostaglandin E2 receptor 3; IL10—Interleukin 10; IL-1β—Interleukin-1 beta. Please note that the up arrow is used to indicate an increase, while the down arrow is used to indicate a decrease.

Significant differences in the incidence of FSs in different populations and geographical regions may indicate genetic involvement in its pathogenesis. In addition, it is now known that having a parent with epilepsy or childhood seizures increases the risk of a child developing FSs [1,15]. The search for possible genetic mutations that lead to an increased risk of seizures has focused on genes for fever response proteins and proteins involved in ion channel function, neurotransmitter release and binding, and vesicular transport (Figure 2).

It has been suggested that body temperature regulation may be different in children predisposed to FSs [71,72]. A genome-wide study supports this notion, identifying two novel loci associated with an altered expression of the *PTGER3* and *IL10* genes [65]. *PTGER3* encodes EP3, one of the four prostaglandin E2 receptors considered to be the major pyrogenic mediator of fever. [73]. The increased expression of the *PTGER3* gene may lead to a more severe febrile response, which, in turn, may increase a child’s risk of developing FSs. *IL10* encodes the anti-inflammatory cytokine IL10, which acts as a central endogenous antipyretic agent in a complex cytokine signaling system [74]. Reduced IL10 levels may also lead to more severe fever, increasing the risk of FSs.

Also, the presence of certain alleles in the genes encoding interleukin-1beta IL-1β and interleukin-1 receptor antagonist IL-1RA also increases the risk of FSs [66,75]. IL-1β is a pro-inflammatory cytokine that triggers a defense response to pathogens, resulting in fever. IL-1RA, which binds to the IL-1 receptor, prevents the activation of intracellular signaling cascades of the pro-inflammatory cytokines IL-1α and IL-1β and plays the role of an anti-inflammatory cytokine. The administration of IL-1RA (anakinra) can attenuate epileptogenesis, as shown in a lithium–pilocarpine model of epilepsy in rats [76]. In vitro experiments have shown that the polymorphism of the genes encoding IL-1β and IL-1RA affects the amount of cytokines produced [77], which, in turn, can lead to a more or less pronounced febrile response. It is true that children with FSs have elevated levels of IL-1β in serum taken within a few hours of the onset of the seizure [75,78].

In addition, mutations in the *GABRG2* gene, which encodes the γ2 subunit of GABA_A_ receptor, have been associated with the development of FSs [67]. *GABRG2* variants are associated with a wide range of epileptic syndromes, from mild cases of FSs and childhood absence seizures to more severe forms of epilepsy such as Dravet syndrome [79,80]. The γ2 subunit is required for the clustering and synaptic localization of GABA_A_ receptors, and the mutant form of this subunit in the receptor pentamer may result in an altered rate of receptor activation [81,82]. Various mutations in the *GABRG2* gene may also alter the kinetics of the GABA_A_ receptor and disrupt its assembly and trafficking [67,83]. Since GABA_A_ receptors mediate most of the fast inhibitory neurotransmission in the central nervous system, a change in their functional properties can lead to the increased excitability of neuronal circuits. Furthermore, it has been demonstrated that elevated temperature (40 °C) leads to the accelerated endocytosis of GABA_A_ receptors containing the γ2 subunit with mutations linked to genetic epilepsy with FSs plus (GEFS+), resulting in a reduction in surface GABA_A_ receptors [84]. This may be one of the causes of the increased susceptibility to FSs observed in individuals carrying these mutations.

Mutations in genes encoding different subunits of the voltage-gated sodium channel have been described in families with GEFS+ [68,69,70]. This syndrome is characterized by the development of recurrent FSs in children, which (unlike typical FSs) continue beyond the age of 6 years. In particular, mutations in the *SCN1A* gene [85], which encodes the α-subunit of the potential-dependent ion channel Nav1.1, and in the *SCN1B* gene [86], which encodes the β1-subunit, have been described in the scientific literature. The occurrence of different mutations can result in either a complete loss of function or a reduction in the functionality of the channel, which may be associated with the clinical severity of the disease manifestation [87,88]. Nav1.1 is expressed in inhibitory interneurons [87,89], and various mutations in *SCN1A* have been demonstrated to reduce sodium current density and action potential frequency in GABAergic neurons [90,91,92,93]. This may result in an imbalance of excitation and inhibition, which is a potential underlying cause of seizures [94]. Nevertheless, a number of *SCN1A* mutations have been linked to an increase in sodium current and a tendency for spontaneous action potential activation in pyramidal neurons of mice aged three weeks in the Dravet syndrome model, which is characterized by severe epilepsy and high lethality [95]. The peak of mortality in this model is observed at the end of the second to the beginning of the third week of life, indicating a correlation between the age-dependent increased excitability of pyramidal neurons and lethality [95]. Furthermore, it was demonstrated that elevated temperatures (40 °C) exacerbate Nav1.1 defects in the presence of mutations, which may explain the heightened susceptibility of individuals carrying these mutations to FSs during fever episodes [96].

Nevertheless, modeling of FSs has demonstrated that the majority of animals can be induced to develop such seizures at an early age, indicating that a genetic predisposition is not a prerequisite. Nevertheless, the presence of the aforementioned genetic mutations may elevate the risk of FSs or result in more severe manifestations.

### 3.2. Malformations of Cortical Development

The most severe and often pharmacoresistant forms of early-onset epilepsy, including a high likelihood of complex FSs, are associated with congenital anomalies in the structure of the cerebral cortex [62,97,98]. Neocortical malformations represent a large and heterogeneous group of neuronal disorders. Developmental abnormalities associated with epilepsy include abnormal cell proliferation or apoptosis (micro- and macrocephaly), abnormal cell migration (lissencephaly, gray matter heterotopia), and abnormal postmigrational cortical development [62].

There are several models of cortical malformations caused by the chemical or physical manipulation of pre- and neonatal animals [99]. In particular, the administration of methylazoxymethanol acetate to female rats on day 15 of gestation impairs the proliferation and migration of brain neurons in the offspring, which subsequently leads to cortical thinning, hippocampal and amygdala dysplasia, and an enlargement of the lateral and third ventricles [100]. The administration of carmustine (1-3-bis-chloroethyl-nitrosourea, BCNU) to female rats on day 15 of gestation results in the disruption of the laminar structure of the cortex and the abnormal morphology of neurons in the offspring [101]. The focal freezing of the newborn rat’s cerebral cortex leads to the development of microgyria in this area [102].

This approach can be used to model a dual pathology: FSs in the context of a cortical malformation. In this case, temporal lobe epilepsy is highly likely to develop [103], making this model a valuable tool for studying epileptogenesis that occurs at an early age.

### 3.3. Sex-Related Characteristics of FSs

Although FSs can occur in both boys and girls, epidemiological studies have identified some differences in the characteristics and risk factors associated with FSs in males and females. These studies have demonstrated a greater prevalence of FSs in boys compared to girls [104]. However, it is important to note that the exact reasons for this male predominance are not well understood. One possible cause is the different timing of the transition from GABA_A_-mediated excitation to inhibition in neurons in males and females [105,106]. During prenatal and early postnatal neurogenesis, GABA acts as an excitatory neurotransmitter, which is essential for neuronal growth and synapse formation [107]. Then, as development progresses, there is a decrease in the expression of the first isoform of the Na-K-Cl cotransporter (NKCC1), which, in the immature brain, ensures the accumulation of Cl^−^ ions in the cell, leading to their release upon the activation of GABA_A_ receptors and, consequently, membrane depolarization. At the same time, the expression of the second isoform of the K-Cl cotransporter (KCC2) increases, which, on the contrary, ensures the removal of Cl^¯^ ions from the cell, resulting in a change in the equilibrium potential of this ion and a change in the function of the GABA_A_ receptor to inhibition [107]. It turns out that this process takes place at different times in female and male rats: in females, increased levels of KCC2 protein in the hippocampus are observed as early as 7 days after birth, whereas, in males, it is not observed until the end of the second week of life [106]. It has been suggested that the explanation for this sex difference may lie in the effect of sex hormones on brain development, including effects on the expression of genes encoding NKCC1 and KCC2 [108].

This suggests that FSs may occur differently in boys and girls due to different levels of excitation and inhibition in the brain in the early postnatal period. This, in turn, can have different effects on the developing nervous system depending on sex. There are few studies on sex differences in the course and outcome of FSs. However, a recent study showed that sexually mature male rats had more severe cognitive impairment than females who had suffered prolonged FSs at an early age [109].

## 4. Models of FSs Ex Vivo

The ex vivo model of FSs involves the study of excitability and cell morphology on slices or cultures of animal brains under hyperthermic conditions, allowing the changes in nervous tissue to be followed directly at elevated temperatures. In this approach, it is important to obtain brain tissue from animals of an appropriate age.

Electrophysiological studies are performed at high temperatures of the perfusion solution with which the brain slice is washed. Under these conditions, when the temperature of the solution is above 38 °C, a single stimulus induces an epileptiform response in brain slices from rats aged 4 to 28 days, while activity develops spontaneously in slices from animals aged 11 to 18 days [110]. Interestingly, in these experiments, the activity persists for an hour or more after the temperature has returned to its initial value (36 °C). No single-stimulus-induced epileptiform activity or spontaneous activity was recorded in slices obtained from animals younger than 4 days old or older than 28 days old [110].

Morphological studies of neural tissue in the in vitro model of FSs are performed on organotypic cultures obtained from the brains of rat pups and incubated at high temperatures. Under these conditions, the expansion of the granular cell layer and its death in the dentate gyrus have been shown, accompanied by the extensive activation of microglial cells [111].

However, when modeling FSs ex vivo, it is important to remember that high temperatures can increase tissue metabolism and reduce oxygen solubility, potentially leading to hypoxia in in vitro preparations where oxygenation may already be difficult. Particularly in electrophysiological experiments, the rate of perfusion solution should be chosen appropriately to minimize the effects of hypoxia, which may confound the results obtained [112].

## 5. Models of FSs In Vivo

### 5.1. Species Used to Model FSs

The initial development of FS models was conducted using rodents, with Sprague Dawley and Wistar rats representing the most prevalent subjects. We did not find any obvious differences in the description of seizures across rat breeds when modeling FSs at 10–11 days of age [26,109,113]; however, it is noteworthy that no direct comparisons have been made.

The accessibility of transgenic and viral genetic technologies, which are frequently employed in murine models, has prompted the adaptation of the rat model for studies in mice, including the assessment of the role of diverse genetic factors in the susceptibility to FSs. It has been demonstrated that C57BL/6J mice, a widely used inbred mouse line, are among the most susceptible to FSs. In this instance, the C3H/HeJ and A/J mouse lines were identified as the most resistant to FSs [30]. It is noteworthy that the A/J mouse line displays a greater susceptibility to chemically and electrically induced seizures in comparison to the C57BL/6J strain [114,115]. This may suggest the existence of disparate initiation mechanisms for distinct seizure types.

Furthermore, FS models have been adapted for use with two-day-old *Drosophila melanogaster* and *Danio rerio* (zebrafish) larvae [31,32]. These model objects offer certain advantages over rodent models [116]. The small size, short life cycle, high reproductive capacity, and ease of maintenance of *Drosophila* and *Danio rerio* make them excellent subjects for genetic studies. In particular, *Drosophila* lines [32] and *Danio rerio* [117], with mutations in the gene of the alpha subunit SCN1A of the potential-dependent sodium channel, have been obtained. In humans, this leads to the development of genetic epilepsy with FSs plus (GEFS+) and Dravet syndrome [32,117]. Furthermore, the use of *Danio rerio* fish as a model subject for neonatal seizures allows for the relatively straightforward conduction of pharmacological studies to evaluate the efficacy of potential anticonvulsant drugs [31,117].

However, it should be noted that fish models have several limitations. The effects of potential drugs may differ significantly between species due to differences in the blood–brain barrier, metabolism, and thermoregulation [118]. Furthermore, the extrapolation of data from *Danio rerio* may result in erroneous conclusions regarding the effects of seizures due to significant differences in the development and morphology of the central nervous system between fish and mammals [119,120]. Nevertheless, the similarities between the principal neurotransmitters, transporters, and receptors of the CNS in fish and mammals render *Danio rerio* an effective model for investigating the etiology of seizure development during the early stages of organism development.

Table 1 provides a summary of representative studies performed on different animal species with a brief description of the FSs.

### 5.2. Hyperthermia-Based Model

Several models, mostly based on hyperthermia alone, have been developed to study the mechanisms and effects of FSs on the developing brain: the hair dryer model [26], the heated chamber model with an infrared lamp [24], the model based on a microwave [25], and the model involving the immersion of animals in heated water heated to 45 °C [27], which can also be referred to as the hot water reflex epilepsy model [123].

The most popular model for FSs, because of its simplicity, is the hair dryer model [26], in which a stream of warm air is generated over the chamber in which the animals are placed. This approach makes it easy to control the chamber temperature and to place and remove animals for frequent monitoring of body temperature. Seizures are modeled at a chamber air temperature of 45–46 °C. Under such conditions, the course of convulsions in 10–11-day-old rats is stereotypical: in the first 10 min, the body temperature rises to 39–40 °C and facial automatisms are observed, often accompanied by unilateral bending of the body, followed by myoclonic twitching of the hind limbs and then clonic convulsions. It should be noted that convulsions induced in rats using this model have been reproduced by several scientific groups [109,113,124], who also note that convulsions develop in an overwhelming number of animals in this model.

Electroencephalogram (EEG) recordings in animals have shown that facial automatisms characterized by forelimb biting and head scratching, observed within the first 10 min, coincide with activity in the amygdala and dorsal hippocampus [26]. An EEG recording of an 11-month-old child having an FS episode showed theta waves recorded in the temporal region [55], and descriptions of the behavior of children with FSs suggest a hippocampal origin of the seizures [56]. This brings this model of FSs closer to the pathogenesis of seizures in children.

The advantage of all hyperthermia-based models is that the timing of the high-temperature exposure can be tightly controlled, allowing the modeling of simple or complex FSs or febrile status epilepticus.

Nevertheless, there are challenges associated with various modeling approaches to hyperthermia. The application of a hot water model to the study of hyperthermia is not feasible for the examination of very small animals. This method entails submerging the animal in water [27], which presupposes the existence of a developed locomotor system. In rats, the final maturation of locomotor functions is observed only by four weeks of age [125]. In addition, different approaches to modeling the FSs show inconsistencies in the main characteristics of seizures. In particular, the use of an infrared lamp has been observed to elicit tonic–clonic convulsions in rats at a rectal temperature of 40–41 °C as early as five days of age [24]. In contrast, the application of hot air has been shown to induce only freezing and rare facial automatisms at a similar rectal temperature in rats at the age of six to seven days. The onset of clonic convulsions has been reported to occur at the age of ten to twelve days in this experimental model [26]. In a model of hyperthermia based on microwave radiation, generalized clonic seizures developed in animals aged 15 days and older, but at a body temperature exceeding 43 °C [50]. Nevertheless, it is challenging to ascertain the precise nature of these discrepancies. Further investigation is required to directly compare the various models of hyperthermia. Different rates of increase in body temperature (both rectal and brain temperature) may be observed, which may be attributed to the differing physical principles of heating utilizing hot air and electromagnetic radiation in the infrared or microwave range. Furthermore, the behavioral responses associated with seizure development may also differ, which would also be evident in direct comparisons within a single study. The behavioral responses may vary depending on the specific parameters employed within a single heating method, such as the wavelength and power of the microwave or infrared radiation utilized. It is therefore possible that the results of studies conducted by different research groups using different models of hyperthermia may be inconsistent or contradictory.

### 5.3. Fever Model

**Figure 3:**
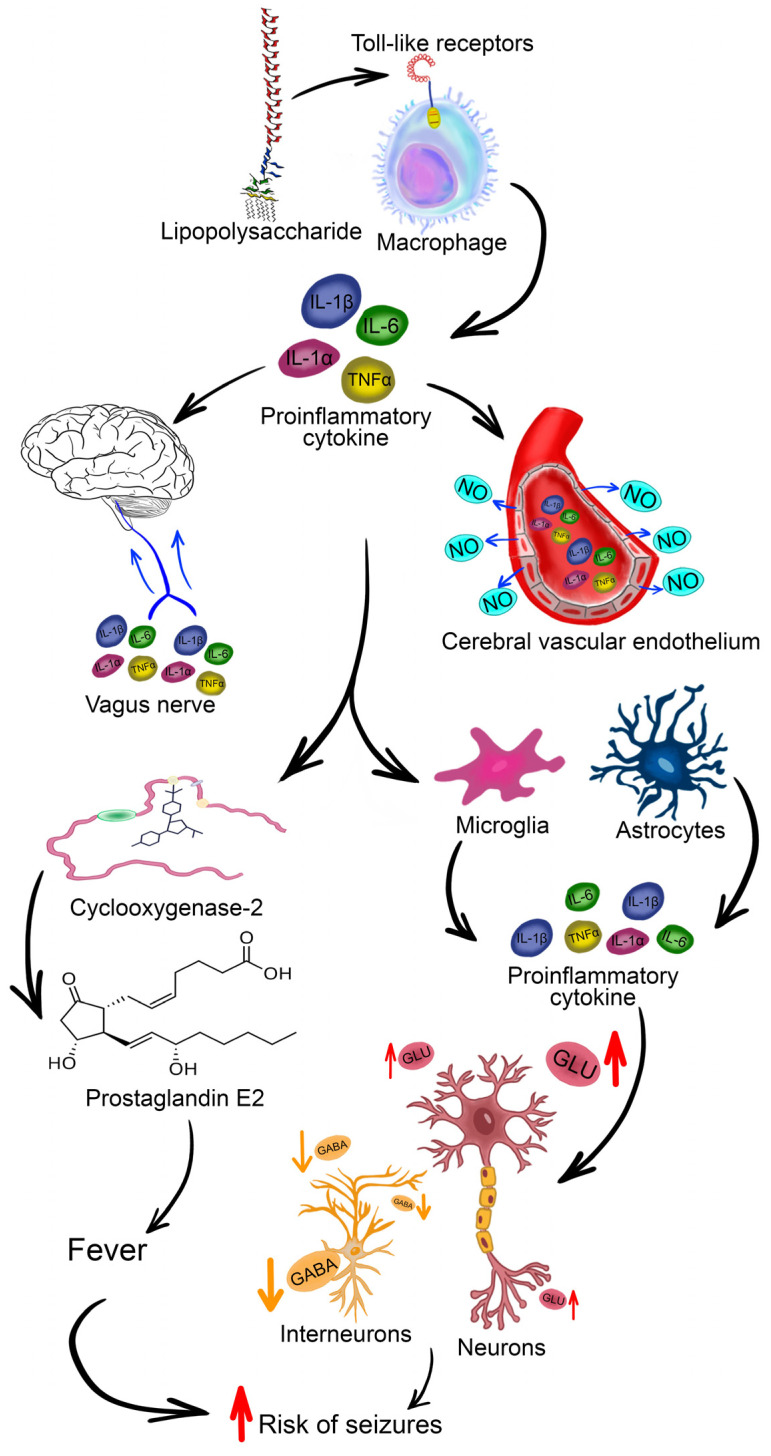
A model of bacterial infection during lipopolysaccharide (LPS) administration. There is minimal direct penetration of LPS across the blood–brain barrier. The effect of LPS on the brain is primarily mediated by pro-inflammatory cytokines, which are secreted by macrophages in response to LPS binding to receptors. Subsequently, the cytokines act via perivascular endothelial cells and afferent nerve fibers in the vagus nerve. This results in the stimulation of the enzyme cyclooxygenase-2, which converts arachidonic acid to prostaglandin E2, thereby inducing fever. Concurrently, glial cells within the brain also generate pro-inflammatory cytokines that regulate neurotransmission and may be implicated in the generation of seizures during fever [128,129,130,131]. The red up arrow indicates an increase and the orange down arrow indicates a decrease.

The vast majority of animal models of FSs are based on hyperthermia rather than fever, which implies the activation of the immune system, although, in children, FSs usually develop in the context of viral or bacterial infection [126]. Inflammatory processes and the release of pro-inflammatory cytokines are thought to be one of the causes of fever [127] and seizure development. In particular, IL-1β, acting through IL-1R1, promotes excitatory transmission by both increasing the conductance of NMDA receptors for Ca^2+^ ions through the phosphorylation of GluN2A and GluN2B subunits [63] and decreasing GABA_A_ receptor-mediated Cl^−^ currents [64] (Figure 3).

Nevertheless, an elevation in body temperature has been demonstrated to result in an augmentation of the concentration of pro-inflammatory cytokines within the blood of animals [132]. The hypothesis concerning the significant function of cytokines in the pathogenesis of FSs is further supported by the observation that the threshold for the onset of seizures induced by hyperthermia is markedly elevated in mice with IL-1R1 receptor deficiency [133]. This also substantiates the notion that hyperthermia per se can lead to the activation of pro-inflammatory pathways.

The administration of lipopolysaccharide (LPS), a component of the cell wall of Gram-negative bacteria, to immature animals does not result in a significant increase in body temperature [28] due to the imperfect thermoregulation in rats at this age [134]. However, even in the absence of convulsions, LPS administration induces alterations in the expression of ionotropic glutamate receptor genes and is accompanied by disturbances in long-term potentiation [135,136]. It is likely that the effect of FSs, occurring in the context of immune system activation, on the developing brain may be more pronounced. Consequently, the use of hyperthermia alone to model FSs may have inherent limitations. However, the current attempt to develop a model of fever-like symptoms using additional immune system activation has yet to yield successful results. A combination of LPS and kainic acid was proposed as a model of FSs [137]; however, this approach does not result in the body temperature reaching the values characteristic of FSs.

### 5.4. Combination Models of FSs

The most approximate model may be a combination of LPS or pro-inflammatory cytokine injections in the context of a hyperthermic model, in which body temperature is raised by hot air [29]. This approach effectively replicates the majority of the underlying mechanisms that occur in actual FSs, resulting in a gradual progression of inflammatory responses, fever, and seizure activity. However, our observations indicate that the concurrent administration of LPS or pro-inflammatory cytokines with a hyperthermic model results in animals that exhibit significantly worse FSs and elevated mortality rates. Consequently, this approach, which may be the most closely aligned with the clinical course of FSs, necessitates the implementation of additional animal care measures and is challenging to employ in a practical setting.

### 5.5. Required Control Groups

**Figure 4:**
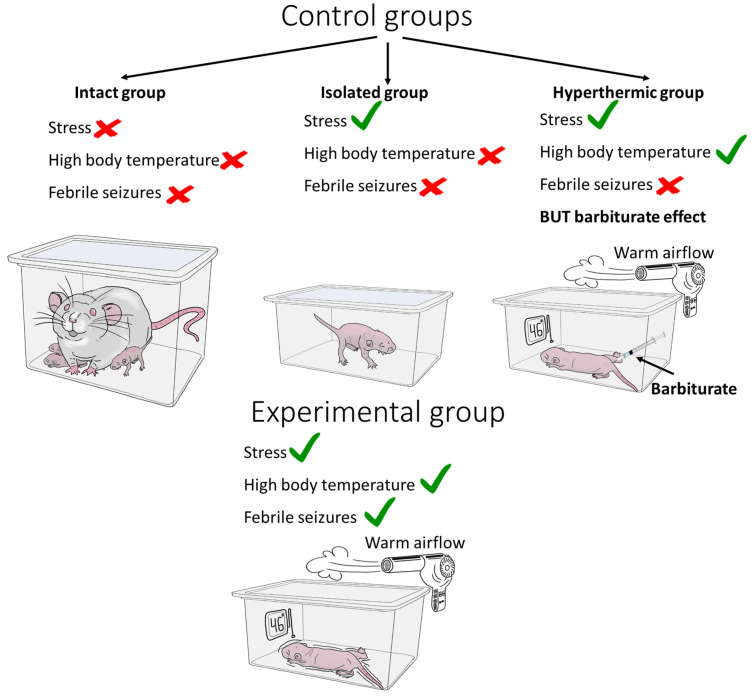
Required control groups. The stress of weaning a pup from the nest can be assessed in isolated animals. The contribution of hyperthermia itself can be assessed by using barbiturates to block seizures. A red cross indicates the absence of a factor and a green tick indicates its presence.

It is incorrect to compare the results obtained in animals subjected to FS modeling with those obtained in intact animals only since the animals are weaned from their dams at the time of modeling, which is a major stress at an early age and may also affect the further development of the nervous system [138,139,140]. Therefore, one of the control groups should be animals weaned from the female for the duration of the FS modeling but kept under normothermic conditions (Figure 4).

The effects of hyperthermia itself, which causes seizures, can also be very important. To assess which effects are specifically due to hyperthermia and which are due to both hyperthermia and FSs developed in this context, some trials have included another control group. Animals in this group have their body temperature raised, but the development of seizures is blocked by the administration of barbiturate [141,142]. However, the mere use of sedatives, including long-acting barbiturates, in immature animals leads to the dysregulation of proteins associated with apoptosis, cell proliferation, and synaptogenesis [143], as well as increased cell death and white matter damage [144,145,146]. In models of status epilepticus, phenobarbital has been shown to exacerbate seizure-induced neuronal damage in rats at an early age [147]. Therefore, short-acting barbiturates (e.g., pentobarbital) should be used in this approach, but there is the possibility that any suppression of neuronal activity in the immature brain, even for a short period of time, may result in impaired cell maturation and synapse formation, and increased apoptosis of immature cells.

## 6. Main Results Obtained in Models of FSs

### 6.1. Morphologic Changes in Neurons

Despite numerous studies, there is still no clear understanding of the morphological changes in the nervous system caused by FSs, which represents an area for further research (Table 2). Clinical studies have indicated that hippocampal edema may occur in children within 48 h of prolonged FSs, with resolution occurring within five days [52]. Studies in animal models of complex FSs indicate that there is no significant loss of hippocampal neurons [148]. The CA1 region of the hippocampus is more susceptible to damage than other regions in the FS model [149]. This may be explained by the later maturation of inhibitory synaptic transmission in CA1 neurons compared to CA3 neurons [150]. However, even in the absence of significant neuronal death, a considerable number of silver-stained “dark” neurons are observed in the hippocampus and amygdala [151]. This indicates that they are functionally damaged [152]. These cells are distinguished by a diminished volume without impairment to the plasma membrane, resulting in ultrastructural compaction (notably, there is a reduction in the volume of the cisternae of the endoplasmic reticulum) [153]. A distinctive attribute of these “dark” neurons is their capacity to recuperate their morphology [154,155,156], which may account for the minimal neuronal loss following FSs.

In the dentate gyrus, where postnatal neurogenesis occurs, one week after FSs, granular cells that appeared after seizures exhibited an increase in dendrite length. Four weeks later, the dendritic tree demonstrated increased complexity. After eight weeks, an additional increase in dendrite complexity was observed, accompanied by an increase in the number of mushroom spines [157]. These morphological alterations in the dendritic structure of granular cells may result in an augmented excitatory signal coming from the entorhinal cortex [158]. Furthermore, an overgrowth of mossy fibers has been documented three months following FSs, with no discernible differences from the control group observed 10 days after seizures [157]. Furthermore, two months following FSs, an increase in F-actin was observed in the presynaptic endings of mossy fiber synapses. This may result in an increase in presynaptic activity and an enhancement of excitatory synaptic transmission between the dentate gyrus and the CA3 region of the hippocampus [159].

The modeling of FSs in older animals (22–30 days) revealed the ultrastructural disruption of synaptic endings in both hippocampal neurons and temporal lobe cortical cells [160,161,162]. Twenty-four hours after the onset of seizures, a series of notable changes were observed in the hippocampal neurons of the CA1 region. These included mitochondrial swelling, degranulation of the rough endoplasmic reticulum, enlargement of the Golgi complex, enlargement of the synaptic gap, reduction in the length of the synaptic active zone, and reduction in postsynaptic density [162]. At three days post-FSs, in addition to the aforementioned ultrastructural disturbances, we also observed swelling of pre- and postsynaptic endings, which exhibited either optically empty fields or minimal residual microfibrillary material and enlarged mitochondria. Furthermore, a reduction in the number of synaptic vesicles was noted in the presynaptic endings [160,161].

**Table 2:** Main findings obtained from FS models following various time periods.

		1 Week After FSs	1 to 4 Weeks After FSs	>1 Month After FSs
Morphology	Neurons	There is minimal cell death in the initial hours following FSs [151] Significant populations of silver-stained “dark” neurons in discrete hippocampal and amygdala regions [151] A decrease in the number of neurons in the hippocampal CA1 region, hilus, and dentate gyrus [149] The ultrastructural disruption of synaptic endings in both hippocampal neurons and temporal lobe cortical cells [160,161,162]	A decrease in the number of neurons in the hippocampal CA1 region [149] Increased dendrite length of granular cells that appeared after seizures [157]	A decrease in the number of neurons in the hippocampal CA1 region [149] Excessive growth of mossy fibers [148] Increase in F-actin in the presynaptic endings of mossy fiber synapses [159] Increased dendritic complexity and number of mushroom-like spines in dentate gyrus cells [157]
	Glial cells	Activation of astroglial cells [163,164] Reduction in astrocytic gap junction coupling, decrease in the level of connexin 43 [121] Edema in astrocytes, damage to the endoplasmic reticulum and mitochondria [165]. Disruption of the blood–brain barrier [166,167] Activation of microglial cells [42,121,164,168]	Activation of astrocytic and microglial cells in the hippocampal CA2 region [169] Impaired of the age-related dynamics of astrocyte maturation [170]	
Synaptic transmission Synaptic plasticity		Decrease in synaptic transmission efficiency and a decrease in calcium conductance through calcium-permeable AMPA receptors [171] Decrease in synaptic transmission efficiency, changes in short-term plasticity in hippocampal CA3-CA1 neurons, and a decrease in the frequency of miniature excitatory synaptic currents [149] Increased GLT-1 levels, reduced glutamate concentration, and unchanged mGlu5R levels in the cortex brain [172]	Increased inhibitory postsynaptic currents in the hippocampus mediated by GABAA receptors [173] Increased Na+/K+-ATPase activity in cortical plasma membranes [174] Decrease in the level of LTP [170,175] Disturbance of age-related changes in the magnitude and character of LTP [170] Heightened desensitization of NMDA receptors [113] Reduction in tyrosine phosphorylation of the GluN2A subunit of NMDA receptors [175] Increase in the threshold for pentylenetetrazol-induced seizures [141] Decrease in GLT-1 levels [172] Increase in mGlu5R levels [172]	Increased expression of the GABAA receptor in granular cells born after FSs in the dentate gyrus [176] Increased Na+/K+-ATPase activity in cortical plasma membranes [174] Decrease in the level of the LTP [170] Increase in the maximal electroshock seizure threshold [149] Increased susceptibility to seizures in rats when kainate was used [177] Electroencephalographically recorded epileptiform discharges in the limbic system [178]
Behavior			Cognitive impairment [179] Depressive-like behavior [180,181] Anxiogenic behaviors [181] Social novelty deficits, repetitive behavior, and hyperlocomotion [169]	Cognitive impairment [109,170,182,183] Depressive-like behavior [180,181,184] Anxiogenic behaviors [181] Social novelty deficits, repetitive behavior [169]

### 6.2. Morphologic Changes in Glial Cells

Currently, there is no consensus regarding the occurrence of astroglial and microglial cell activation following FSs. Modeling febrile status epilepticus in 11-day-old rats, astroglial cell activation was observed in the CA3 region of the hippocampus and in the dentate gyrus 6 h after seizures [163] and in the CA1, CA3 regions, and hilus of the hippocampus 24 h after seizures [163,164]. Neither in the rat model of FSs 2 days after seizure nor in the mouse model after 5 days was astrogliosis detected in the hippocampus [121,170]. The evaluation of the area occupied by astrocytes in the CA1 and CA3 regions of the hippocampus 2 days and 11 days after FSs revealed that the age-related dynamics of astrocyte maturation is impaired in animals after seizures, which is accompanied by impaired synaptic plasticity [170]. In FS modeling of 14–15 day old mice 5 days after seizures, a significant inhibition of inter-astrocytic gap junctions in the hippocampus is observed, which is accompanied by a decrease in the level of connexin 43, one of the gap junction proteins [121]. The disruption of astrocytic gap junctions can result in the impaired clearance of excess potassium ions and glutamate from extracellular space, which may contribute to increased neuronal excitability [185,186]. The disruption of ion and molecule distribution through the astrocytic network can result in cell edema [186], as evidenced by an ultrastructural study of hippocampal and temporal lobe cortex astrocytes in a rat model of FSs three days after seizures [165]. In addition to edema, degenerative changes in astrocytes have been described, with the main manifestations being damage to the endoplasmic reticulum and mitochondria [165]. These changes can lead to disturbances in some biochemical processes, such as abnormal protein synthesis or the inhibition of oxidative phosphorylation.

At one and three days following FSs, rats exhibit a disruption of the blood–brain barrier [166,167], as evidenced by weakened and destroyed dense intercellular endothelial connections and frequent complete occlusion of the capillary lumen, caused both by endothelial cell damage and external pressure from markedly enlarged perivascular astrocytic processes [166].

Extensive microgliosis was demonstrated by in vitro experiments on organotypic cultures of hippocampal slices, which were subjected to heat shock [111]. The results of some in vivo experiments align with this finding. The activation of microglial cells is observed in the rat hippocampus 24 h after FSs [42], with the most significant changes occurring in FS modeling at 15 days of age in comparison to the microglial response in FS modeling at 5, 10, or 20 days of age. These changes include an increased number of microglial cells and morphological alterations in individual cells, such as the emergence of short, thick outgrowths and a distinct large soma [42]. In mice aged 14–15 days, microglial cell activation is observed within 1 to 7 days after seizures [121,168]. However, when modeling FSs on animals aged 10–11 days, no increase in the total number of microglial cells is observed. However, there is a significant increase in the proportion of activated microglia with an amoeboid shape [164]. Recently, microglial cell activation has been considered as two polar states: classical (the M1 phenotype) or alternative (the M2 phenotype). The M1 phenotype has been demonstrated to release pro-inflammatory factors and free radicals, which have been shown to impair the repair and regeneration of brain tissue. In contrast, M2 microglia facilitate brain repair and regeneration by enhancing phagocytosis, releasing trophic factors, and eliminating inflammation [187,188]. In a mouse model of FS, it has been demonstrated that hyperthermia activates microglial TRPV1, which subsequently suppresses microglia activation via the M2 pathway [168]. The inhibition of microglial polarization through the alternative pathway can result in the predominance of the M1 phenotype and the subsequent release of pro-inflammatory cytokines, including interleukins-1β, interleukin-6, and tumor necrosis factor-α (TNFα), whose increase in the brains of experimental animals after FS has been confirmed in several studies [42,164,169,189].

### 6.3. Changes in Neuronal Properties and Synaptic Transmission

The use of an ex vivo model of FSs allows us to directly record changes in the biophysical properties of neurons and synaptic transmission in brain slices as temperature rises, which may provide valuable information about the causes of seizure activity and the search for targets to stop it. Thus, hippocampal slices from 13- to 16-day-old mice showed that the high-temperature bath solution (41 °C) led to increased excitability, decreased input resistance, and spontaneous activity of both excitatory pyramidal neurons and inhibitory interneurons in the hippocampus [190]. The increase in neuronal excitability at elevated temperatures is associated with a calcium current mediated by the Ca_v_1.2 subunit of L-type calcium channels. At the same time, the selective L-type calcium channel blocker nimodipine reduces the frequency and duration of seizures in animals in an experimental model of FSs in vivo [60]. Using this model, it has also been shown that increasing temperature increases the sodium current through the non-selective cation channel NALCN, which also leads to the depolarization of the nerve cell membrane [60].

The use of in vivo models of FSs makes it possible to assess the short- and long-term effects of FSs. Many studies have focused on neuronal excitability and synaptic transmission at different times after FSs. Immediately after FSs, a decrease in synaptic transmission efficiency and a decrease in calcium conductance through calcium-permeable AMPA receptors are observed in rat hippocampal neurons [171]. This is thought to prevent excessive excitotoxicity, and no significant neuronal death is observed in this model. Two days after seizures in rats, a decrease in synaptic transmission efficiency, changes in short-term plasticity in hippocampal CA3-CA1 neurons, and a decrease in the frequency of miniature excitatory synaptic currents in hippocampal CA1 field neurons were observed. In general, these data indicate a decrease in the probability of mediator release [149]. Furthermore, an additional neuroprotective mechanism has been delineated in the context of augmented glutamate transporter 1 (GLT-1) levels following hyperthermia-induced seizures [172]. This may contribute to a reduction in the concentration of glutamate within synaptic clefts [191], which was validated in the rat cerebral cortex two days following FSs [172].

One week after seizures in rats, the stimulation of Schaffer collaterals with a single stimulus resulted in a decrease in the amplitude of population spikes in CA1 neurons as a result of increased inhibitory postsynaptic currents in the hippocampus mediated by GABA_A_ receptors [173]. However, when Schaffer collaterals are stimulated with a series of stimuli, epileptiform activity develops only in slices obtained from animals one week after FSs compared to the control, indicating increased excitability of hippocampal neuronal circuits [177]. This contradiction can be explained by the fact that increased inhibition can reduce activity in response to a single stimulus, but when stimulated by a series of pulses, increased GABA_A_ conductance leads to an accumulation of Cl^−^ in neurons, a strong activation of the KCC2 transporter, and an increase in K^+^ ions in the extracellular space, resulting in a shift in the neuronal resting potential towards depolarization and increased cell excitability [192,193]. An increased expression of the GABA_A_ receptor in granular cells born after FSs has also been demonstrated in the dentate gyrus [176]. In addition, increased Na^+^/K^+^-ATPase activity was found in rat cortical membranes 20 days and 2 months after hyperthermia-induced seizures, which may also represent a compensatory mechanism for a decrease in the extracellular concentration of K^+^ ions and, consequently, a decrease in the excitability of cortical cells [174]. However, 20 days after the seizures, an increase in metabotropic glutamate receptor 5 (mGluR_5_) levels was observed [172]. The activation of mGluR_5_, a member of group I metabotropic glutamate receptors, leads to cell depolarization, increased neuronal excitability, and the modulation of NMDA receptors [194,195]. Although the causal relationship between group I mGluRs and susceptibility to seizures has not yet been fully established, high expression of mGluR_1_ and mGluR_5_ is considered a potential mechanism of epileptogenesis [196]. Consequently, the increase in mGluR_5_ levels observed in the FS model may lead to an elevated risk of recurrent seizure episodes.

### 6.4. Changes in Synaptic Plasticity

In examining the effects of FSs on synaptic plasticity, a number of studies have reported conflicting results: increased long-term potential (LTP) and decreased long-term depression (LTD) in CA3-CA1 synapses of the hippocampus 1 month after complex FSs [197]; conversely, decreased LTP in animals 11 days and 1.5 months after complex FSs [113,170]; and, in addition to decreased LTP, increased LTD 1 month after complex FSs [175], indicating the need for additional studies. In examining LTP in rats of varying ages, it was shown that FSs might impede the functional maturation of the hippocampus. This is because rats at three weeks of age exhibit an unstable LTP [170], a phenomenon observed in younger animals (less than two weeks old) [198,199,200,201].

One potential mechanism underlying the impairment of synaptic plasticity following complex FSs may be a reduction in the tyrosine phosphorylation of the GluN2A subunit of NMDA receptors [175]. Additionally, it was demonstrated that the reduction in LTP observed in rats following FSs may be attributable to a heightened desensitization of NMDA receptors [113]. The absence of changes in NMDA receptor subunit composition after FSs [113,175] and the restoration of the level of LTP to control values with the NMDA receptor glycine site coagonist D-serine [113,170] indicate that the observed pronounced desensitization may be due to insufficient activation of the NMDA receptor glycine site [202,203]. Furthermore, the restoration of LTP levels in the presence of D-serine may suggest impaired interactions between neurons and glial cells, as astrocytes have been demonstrated to regulate NMDA-dependent plasticity by the Ca^+2^-dependent release of D-serine [204].

### 6.5. A Predisposition to Recurrent Seizures and Epilepsy

When evaluating the seizure threshold in rats after FSs, mixed results were obtained. An increase in the threshold for pentylenetetrazol-induced seizures has been shown [141], along with an increase in the maximal electroshock seizure threshold [149]. Meanwhile, other authors have demonstrated an increased susceptibility to seizures in rats when kainate was used [177], in addition to electroencephalographically recorded epileptiform discharges in the limbic system [178]. Such differences in results could arise, firstly, from the different ages of seizure susceptibility assessment. Thus, an increase in the seizure threshold has been shown in younger animals (<2.5 months). Secondly, all the studies used different approaches to study the threshold of seizure development; in particular, the use of pentylenetetrazole, which is an antagonist of GABA_A_ receptors, resulted in an increase in the threshold of seizure development, while the use of kainic acid, which is an agonist of kainate and AMPA receptors and increases excitatory transmission, led, on the contrary, to a decrease in the seizure threshold. These data show the need for a more detailed study of the effect of FS on the probability of recurrent episodes of seizures and epilepsy.

### 6.6. Behavioral Disorders After FSs

A number of studies have described memory impairment in children following prolonged exposure to FSs [205,206]. A substantial body of experimental evidence from animal models of FSs has also demonstrated cognitive impairment in both immature [179] and adult [109,170,182,183] animals. This is evidenced by impaired spatial memory in Morris and Barnes mazes [170,179,182,183], reduced latency period in the inhibitory avoidance task [182], and increased electrocution in the active avoidance test [109].

In addition to cognitive impairment in animals, depressive-like behavior was noted in models of FSs, manifested by decreased sucrose preference and increased immobility during the forced swim test in rats at 37 and 60 days of age [180]. However, it should be noted that in this study, febrile convulsions were modeled by the administration of LPS and kainic acid. This leads to the development of convulsions in the context of the activation of inflammatory cascades, although no significant increase in body temperature necessary for the development of febrile convulsions is observed in this model. However, other hyperthermia-based models have also shown depressive-like behavior and increased levels of anxiety-like behavior in adult animals [181,184]. A recent study in an FS model described animal behavior similar to autism spectrum disorders, including social novelty deficits, repetitive behavior, and hyperlocomotion [169].

Thus, to date, there is considerable evidence that FSs adversely affect the development of the nervous system, leading to a wide range of different neurological disorders.

## 7. Conclusions: Limitations and Future Directions

As FSs remain one of the most common childhood neurological disorders, it is important to find ways to predict its development, suppress seizure activity, and prevent the development of subsequent neurological disorders. These questions can only be answered by a combination of long-term clinical observation of patients and studies in experimental animal models. The advantage of animal models over clinical studies of FSs is the ability to either exclude the influence of any factors, such as pre-existing CNS pathology, genetic predisposition, or drug side effects, or to simulate dual pathology. In addition, working with experimental models allows strict control of the timing of hyperthermia and the duration and nature of the seizures, which can be a key indicator for assessing the results.

As is the case with numerous animal models of diverse diseases, the FS model has certain limitations. The main challenges associated with modeling FSs in animals and the subsequent evaluation of the outcomes are due to the fact that FSs manifest at an early age when the maturation of the nervous system is actively occurring. However, the development of different brain regions in humans and mice or rats occurs at varying rates, and these processes are not necessarily parallel in humans and rodents. This discrepancy may result in erroneous conclusions when attempting to extrapolate findings from rodent models to humans.

Another limitation is the difficulty of modeling the cause of fever and the inflammatory response, which is the most common cause of hyperthermia and FSs in children. Currently, models based on hyperthermia are most commonly used, although it is known that temperature elevation itself also leads to the release of pro-inflammatory cytokines [132,133]. However, in this case, hyperthermia occurs first, followed by an inflammatory response of the nervous system, whereas, in clinical cases, inflammation develops first, then fever, and then seizures. This initial inflammation already affects synaptic transmission [63,64], so models based on hyperthermia alone may not fully reflect the course of FSs in children.

Nevertheless, despite the aforementioned limitations, the existing animal models continue to represent a valuable tool for the study of FSs. One of the most pressing areas for future research on animal models of FSs is the identification of therapeutic agents for the management of FSs in the context of fever. To date, clinical practice has demonstrated that the use of antipyretic drugs, including ibuprofen, diclofenac, and acetaminophen, does not prevent the development of FSs in children. Similarly, anticonvulsants, such as phenobarbital, primidone, and valproic acid, while reducing the risk of recurrent seizures, have side effects that outweigh the benefits [207]. In vivo experiments in rats have demonstrated that nimodipine, a calcium channel blocker, can reduce the frequency and duration of FS in rats. However, it should be noted that high doses of nimodipine can cause side effects that may be related to the inhibition of calcium currents in the cardiovascular system [60].

In mutations of the *SCN1A* gene associated with more severe neurological diseases, such as GEFS+ and Dravet syndrome, which are also characterized by FSs, GABAergic neurotransmission is impaired, resulting in an increased excitability of neuronal circuits. There are indications for the use of benzodiazepines (intravenous lorazepam, rectal diazepam, or intranasal midazolam) to treat febrile status epilepticus or prolonged FSs in clinical practice [208], but their use to reduce relapse rates is not recommended because the side effects outweigh the potential benefits [209]. However, low doses of clonazepam, an allosteric modulator of GABA_A_ receptors, were able to correct behavioral abnormalities, including abnormal social behavior and fear memory deficits, in a genetic mouse model of Dravet syndrome *Scn1a^+/−^* [210], indicating the possible use of low doses of benzodiazepines as a pharmacological intervention to alleviate cognitive deficits in mutations of the *SCN1A* gene.

The advent of novel transgenic animal models and the advancement of viral genetic constructs may also facilitate the development of innovative genetic therapies. To date, several mouse models of Dravet syndrome [211] and GEFS+ [212] with various mutations in the *Scn1a* gene, which encodes the α-subunit of the Na_V_1.1 potential-dependent ion channel, have been described. A number of models were created by introducing a point mutation [212,213], resulting in a less severe disease phenotype. However, a predisposition to FSs was observed in such mice [213]. The injection of an adenoviral vector carrying *SCN1A* cDNA into the brains of mice with a missense mutation in *Scn1a* resulted in the expression of functional Na_V_1.1, thereby increasing the threshold for the development of hyperthermia-induced seizures [214]. The use of dCas9 for gene editing has been observed to enhance the transcription of the *Scn1a* gene in the mouse brain, also resulting in a higher threshold for the development of hyperthermic seizures [215,216]. The described different mutations in the *GABRG2* gene encoding the γ2 subunit of the GABA_A_ receptor, which occur in GEFS+, may also lead to the development of a genetic therapy that could target *GABRG2* [217].

Chemogenetics, which has already been tested in models of epilepsy [218,219,220], may be another potential avenue for seizure control. Designer receptors exclusively activated by designer drugs (DREADD) represent a powerful approach to the remote and short-term manipulation of cellular activity. The main advantage of this approach has been cited as the fact that DREADD receptors respond exclusively to the synthetic ligand clozapine-N-oxide, which was previously considered to be an inert compound with no pharmacological activity against non-DREADD targets [221]. However, a recent study has challenged this assumption by showing that clozapine *N*-oxide undergoes reverse metabolism to clozapine, which is an atypical antipsychotic and causes behavioral changes [222]. This indicates that the use of this method to attempt to treat FSs should only be used after more specific ligands for DREADD have been found, as any side effects may be dangerous to the immature brain.

Although gene therapy has recently made significant progress, its use in the management of FSs in clinical practice can be challenging. It is essential to have precise indicators that suggest a high likelihood of FSs in a febrile child to justify the utilization of gene therapy. Gene therapy entails modifying the delicate equilibrium between excitation and inhibition in the brain, which has the potential to influence the maturation of the nervous system at an early developmental stage. It is crucial to ascertain whether the potential benefits of this approach outweigh the probable consequences of modifying synaptic transmission during brain development. It is therefore more probable that gene therapy will be employed in a clinical setting for more severe neurological disorders, such as Dravet syndrome or genetic epilepsy with FSs plus, rather than for the control of single episodes of FSs.

## Figures and Tables

**Table 1:** The animals employed as subjects in FS models, along with their principal characteristics of seizures.

Model Organism	Line/Breed	Age	The Characteristics of the Convulsive Episode	Source
Rat	Sprague Dawley	P6-P7	Body temperature rises to 40 °C. Freezing, rare facial automatisms	[26]
	Sprague Dawley Wistar	P10-P12	Body temperature rises to 39–40 °C. Facial automatisms, often accompanied by unilateral bending of the body. Myoclonic twitching of the hind limbs. Clonic convulsions.	[26,109,113]
	Wistar	P20	Threshold temperature 44 °C. Generalized clonic convulsions. The duration of convulsions is significantly longer than in rats of other ages.	[50]
Mouse	C57BL/6J	P10	Similar to the course of FSs in rats at the age of P10	[121]
	Transgenic mice with human GFAP (hGFAP) promoter-controlled expression of EGFPP (based on the FVB/N line)	P14-P15	Several episodes of sudden cessation of activity and prolonged immobility with reduced responsiveness. Facial automatisms (chewing). Clonic or tonic–clonic seizures are not observed.	[121,122]
*Drosophila*		2-day-old fly	Brief leg twitches, followed by failure to maintain standing posture, with wing flapping, leg twitching, and sometimes abdominal curling.	[32]
*Danio rerio*		larval aged 3 to 7 days post-fertilization	After implantation of a glass microelectrode into the forebrain of the larva, abnormal electrographic convulsive activity is recorded when the temperature in the bath where the larvae are kept rises.	[31]

## Data Availability

Not applicable.
